# Dynamic Cooperative Communications with Mutual Information Accumulation for Mobile Robots in Industrial Internet of Things

**DOI:** 10.3390/s24134362

**Published:** 2024-07-05

**Authors:** Daoyuan Sun, Zefan Liu, Xinming Zhang

**Affiliations:** 1 Department of Computer Information Engineering, Anhui Vocational and Technical College of Industry and Trade, Huainan 232007, China; daoyuansun@ustc.edu.cn; 2 School of Computer Science and Technology, University of Science and Technology of China, Hefei 230027, China; lzf1111@mail.ustc.edu.cn

**Keywords:** cooperative communication, industrial Internet of Things, mobile robot, mutual information accumulation, rateless code, wireless sensor networks

## Abstract

Mobile robots play an important role in the industrial Internet of Things (IIoT); they need effective mutual communication between the cloud and themselves when they move in a factory. By using the sensor nodes existing in the IIoT environment as relays, mobile robots and the cloud can communicate through multiple hops. However, the mobility and delay sensitivity of mobile robots bring new challenges. In this paper, we propose a dynamic cooperative transmission algorithm with mutual information accumulation to cope with these two challenges. By using rateless coding, nodes can reduce the delay caused by retransmission under poor channel conditions. With the help of mutual information accumulation, nodes can accumulate information faster and reduce delay. We propose a two-step dynamic algorithm, which can obtain the current routing path with low time complexity. The simulation results show that our algorithm is better than the existing heuristic algorithm in terms of delay.

## 1. Introduction

The industrial Internet of Things (IIoT) is able to effectively promote industrial production and has been studied recently [1,2]. In the IIoT, mobile robots play an important role, which are generally used in many aspects of the IIoT, such as factory damage monitoring, data collection, cargo handling, etc. As the concepts of Industry 4.0 and 5.0 have been proposed and widely recognized in recent years [3,4,5], the in-depth participation of various kinds of intelligent mobile robots in industrial manufacturing will further improve production efficiency [6]. Related enabling technologies, such as digital twins [3,5], edge computing [7,8], and other technologies, also put forward higher requirements for communication between robots and the cloud.

When mobile robots move in the factory, they need to upload the collected information to the cloud, and the cloud needs to transmit instructions to them. For example, Karthikeyan et al. [9] implemented a mobile robot for fire alarms. It carries a variety of sensors such as temperature, smoke, chemical gases, etc., to quickly determine whether it is a false alarm or a real fire and notify evacuees. Li et al. [10] proposed a way to use information collected by sensors in the field to navigate mobile robots, especially data that require large sensors to easily collect. Wan et al. [11] pointed out that the mobile robot itself has limited computing power. According to [11], robots could solve the problem of insufficient computing power through handing over the computing task to the cloud. Taking a mobile robot for cargo handling as an example, the mobile robot needs to transmit information such as its own status and location information to the cloud in a timely manner, and the cloud schedules tasks for multiple mobile robots to maximize work efficiency. These results illustrate the necessity of mobile robots communicating with the cloud. In order to achieve this goal, data transmission between the mobile robot and the cloud needs to meet a sufficiently low latency and high reliability [12]. In industrial scenarios, the communication delay that mobile robots can tolerate is 40–500 ms [13]. For the above requirements, 5G URLLC and mMTC types of services already provide high-quality solutions [14,15,16]. However, it may introduce additional deployment costs. This paper will try to make full use of the wireless sensor nodes and sink node commonly deployed in industrial scenarios to provide communication between mobile robots and the cloud. Since this paper deals with the IIoT rather than the Internet of Robot Things (IoRT) [17], it is assumed that the wireless sensor nodes are not be dedicated to providing perception data or services for mobile robots. When the mobile robot is within the transmission range of the sink node wired with the cloud, it can directly transmit data to the sink node. When the mobile robot moves outside the one-hop communication range of the sink node, or there is an obstacle between the mobile robot and the sink node, it is necessary to use relays for multi-hop transmission. In this paper, we assume that there is always a valid path to the sink.

**Figure 1:**
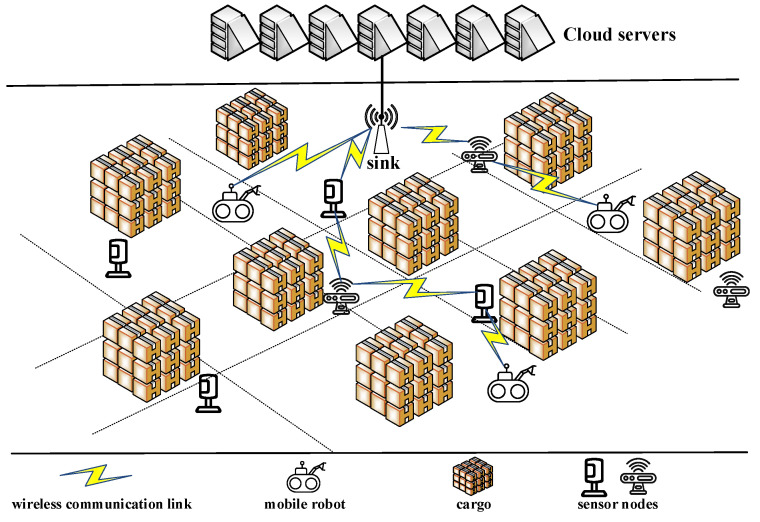
Mobile robots in IIoT.

**Figure 2:**
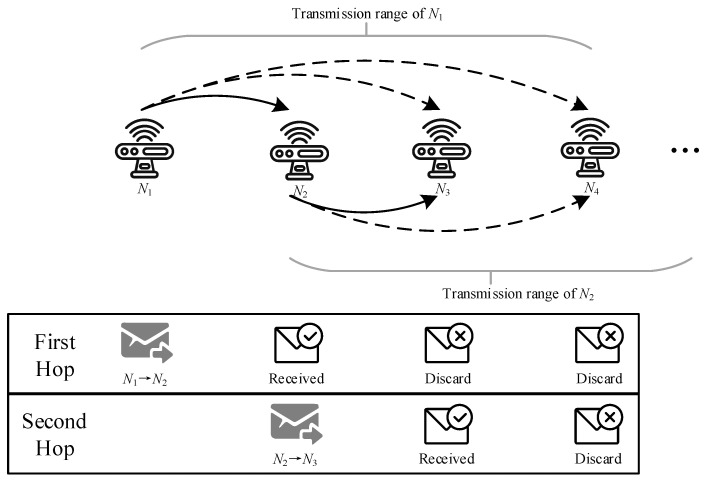
Conventional wireless transmission.

As shown in Figure 1, in the environment of the IIoT, there are many fixed sensor nodes, which can be used as relay nodes for communication between sink and mobile robot. These sensor nodes monitor the environment of the factory in various places, and form wireless sensor networks (WSNs) to transmit the generated data to the sink node. Li et al. [10] used the environmental information monitored by WSNs to navigate the robot. This illustrates the feasibility of using WSNs as relays between mobile robots and sink nodes. For conventional WSN multi-hop routing, it is essential to find a balance between the number of hops and the transmission distance of each hop, because the end-to-end delay will increase as the number of hops increases, while reducing the number of hops means that the average distance of each hop becomes longer, which will reduce the probability of successful transmission, lead to unnecessary retransmissions, and then introduce more delays. This contradiction will be more obvious under complex channel conditions in industrial scenarios. In addition, considering the dense deployment of nodes, during the multi-hop forwarding process, nodes can not only receive broadcasts from the previous hop node, but also receive broadcasts from other nodes (such as nodes in the previous few hops). In conventional WSN routing, the above two types of information are usually discarded directly, as shown in Figure 2.

To achieve real-time communication between robots and the cloud, there are two major challenges: the end-to-end delay and the time complexity of the routing algorithm. For the former challenge, the time delay on a single transmission cannot be too long, and for the latter challenge, the mobile robot needs to quickly find a new routing path after moving. Existing research does not solve these two challenges well. In this paper, the communication between the robot and the cloud is modeled as a multi-hop cooperative communication problem by using WSNs as relays, and we propose a dynamic and efficient cooperative transmission algorithm to solve these two challenges.

**Figure 3:**
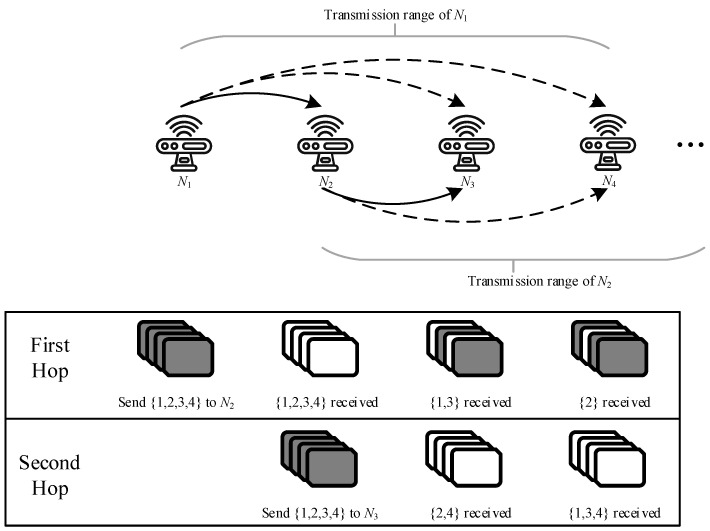
Transmission with mutual information accumulation.

To meet the end-to-end delay requirements of mobile robots, cooperative transmission with mutual information accumulation has been introduced. In conventional wireless transmission of hop-by-hop transmission, a node only receives information from the previous hop node and transmits it to the next hop after decoding. However, cooperative transmission with mutual information accumulation allows different nodes to broadcast independent packets to accumulate information more quickly to complete decoding [18]. Mutual information accumulation uses rateless codes, so that nodes can decode the original data through information received from different sources. In this case, the broadcasting characteristics of wireless transmission have been fully utilized. In conventional wireless transmission, a node can only receive and decode information from one node. Therefore, in a node’s broadcast, only one receiver can successfully decode and act as a relay, while other nodes must drop all the information they received. Unlike automatic repeat request (ARQ), rateless coding is a forward error correction (FEC) coding scheme where the sender only needs to keep sending coded packets until the receiver accumulates enough packets and decodes them correctly, and then the sender is told to stop sending. If the receiver fails to receive a coded packet correctly, it just needs to receive the next packet normally without requesting retransmission until it receives enough packets for decoding. With the help of mutual information accumulation, when a node performs broadcasting, all its neighbors are able to accumulate information for subsequent decoding. The routing path of transmission with mutual information accumulation can be represented by the node’s decoding order. In the rest of this paper, we will mix the concepts of routing path and decoding order. As shown in Figure 3, there is no doubt that the mutual information accumulation has a lower delay than conventional wireless transmission on the same routing path because the information accumulated in advance is used.

Since WSNs are fixed, their topology can be viewed as static over a relatively long period of time. Based on this observation, we have designed an adaptive two-step routing algorithm. The first part is the static part, in which the routing path and delay from the sink node to all nodes in WSNs are calculated through the modified Dijkstra algorithm. This part of the algorithm will only be performed once in a relatively long time, and the result can be saved on all nodes. In the second part, the dynamic part will be frequently executed once the mobile robot moves. Obviously, there are two communication cases in total, which are transmissions from the sink to the mobile robot and from the mobile robot to the sink. In the former case, we searched for neighboring nodes of the mobile robot and used the routing path with the shortest delay as the current routing path. For the latter case, we use a distributed greedy algorithm to always send information to the node with a shorter delay from the sink.

In summary, to realize communication between cloud and mobile robots, we propose a dynamic cooperative transmission algorithm with mutual information accumulation. The main contributions of this paper are as follows:

- Existing research on mutual information accumulation only applies to WSNs with fixed node deployments and does not consider the issue of node mobility. This paper designed the routing algorithm based on the mobility of mobile robots, which was not available in previous scenarios.
- The fixed deployment node routing algorithm proposed in this paper introduces cooperative communication based on mutual information accumulation into WSN scenarios, and simulation experiments showed that it can reduce the delay of multi-hop relays.
- The dynamic path algorithm and distributed heuristic algorithm proposed in this paper can dynamically adapt to the new position of the mobile robot without frequently updating routing information, which could significantly reduce the time complexity of the routing algorithm.

The rest of this paper is organized as follows. In Section 2, we show related work. In Section 3, we describe system models and assumptions, and analyze optimization problems. In Section 4, we detail our dynamic collaborative routing algorithm and analyze its complexity. Simulation and numerical results are presented in Section 5. Finally, we conclude this paper in Section 6.

## 2. Related Work

Existing research on mobile robots has mainly focused on the physical pathfinding and scheduling of mobile robots. A context-aware cloud robotics (CACR) architecture was designed in [11] to save energy and improve the material handling capabilities of mobile robots. Li et al. [10] used WSNs to find a barrier-free road for robots to eliminate the burden of mobile robots carrying large sensors. These studies did not solve the communication problem between mobile robots and the cloud. Wichmann et al. [19] proposed a method of using multiple robots to form a communication path to ensure communication between the sink and the robot at the event site. However, they did not make full use of the sensor nodes in the environment, making it necessary to process an event with multiple robots. Ref. [20] studied cooperation strategies between unmanned aerial vehicles (UAVs) to reduce packet retransmission probability by reducing unnecessary automatic repeat requests (ARQs). Kondo et al. [21] proposed and implemented a WiFi-based mobile robot communication system to achieve remote operation of the mobile robot. However, neither of them can be applied to IIoT scenarios.

Rateless codes, or fountain codes, can be decoded after receiving any sufficient amount of information. The performance of the rateless code over a fading channel was confirmed in [22,23]. Molisch et al. [24] first proposed cooperative transmission with mutual information accumulation using rateless code. Utilizing the characteristics of the rateless code, mutual information accumulation can receive mutually independent packets from different nodes, thereby completing decoding faster than energy accumulation. Guo et al. [25] proposed a multi-hop relay software distribution protocol based on rateless coding, which dynamically encodes data packets according to the state of the network to achieve efficient software distribution in IoT networks. Draper et al. [26] proposed an optimal cooperative transmission algorithm with mutual information accumulation, which optimized the decoding order and relay subset alternately. Urgaonkar et al. [27] proved that under the condition of time division multiplexing, the optimal transmission sequence of any transmission set can be obtained by the greedy algorithm. Therefore, the alternate optimization of the transmission set and the decoding order becomes the search for the optimal transmission set. However, the search for the optimal transmission set still cannot be completed in polynomial time, so they also proposed two heuristic algorithms based on Dijkstra. Shakiba-Herfeh et al. [28] considered the optimal algorithm and heuristic algorithm, and found that the node energy may be exhausted in the middle of transmission. However, none of the above algorithms considers the situation where one of the sender or receiver is moving. During the movement of the mobile robot, it is necessary to quickly obtain a high-quality transmission path.

## 3. System Model and Assumptions

### 3.1. System Model

As shown in Figure 1, the system consists of a sink node *S*, sensor nodes *N*, and mobile robots *M*. The sink node is fixed in the field and directly connected to the cloud. The information transmitted to the sink node will be processed by the cloud or edge node immediately. The information that the cloud needs to send must also be sent from the sink node. We refer to the mobile robots as the source nodes. Considering that there may be more than one mobile robot in an actual scenario, and there may be competition and interference between multiple mobile robots, this makes the multi-hop routing algorithm based on mutual information accumulation from multiple sources to sink have high time complexity. We tried to reasonably simplify the scenario and obtain a simple, effective, and scalable solution by adopting a divide-and-conquer strategy. Therefore, without loss of generality, we abstract the problem studied in this paper into a multi-hop relay routing problem between a movable source node and a fixed deployment sink node.

Sensor nodes are uniformly randomly distributed in the field. The topology of WSNs can be represented by a graph G={V,E}, where *V* is the set of sensor nodes and *E* is the neighbor relationship between sensor nodes. Each node knows the information of neighbor nodes and the quality of the link to it. The sink node knows the global link information and can make a global plan for the routing path. We assume that there are fixed or mobile wireless chargers that can charge sensor nodes to ensure that all nodes will not run out of energy. This is reasonable because many references, such as [29,30], provided specific wireless charging schemes. We assume that the sensors participating in forwarding have the required energy supply to achieve a reasonable simplification of the problem studied. When a node starts transmitting, all neighbor nodes within its transmission range can accumulate information.

We assume that the channel quality between WSNs is relatively stable. The sink node can collect link quality information from all links between WSNs and use it until the next collection. After the sink node has calculated the routing table of the current WSNs, all sensor nodes and mobile robots can know and use the results. The routing algorithm inside WSNs can have relatively high complexity because its calculation results can be used for a long time.

The mobile robot moves in the field according to its task. It needs to hand over computing tasks to the sink node and accept instructions from the sink node. Therefore, the mobile robot needs to communicate frequently with the sink node. When the robot moves, it knows the changes in neighboring sensor nodes. In addition, the sensor node can also know that the mobile robot has arrived nearby.

### 3.2. Transmission Model

**Figure 4:**
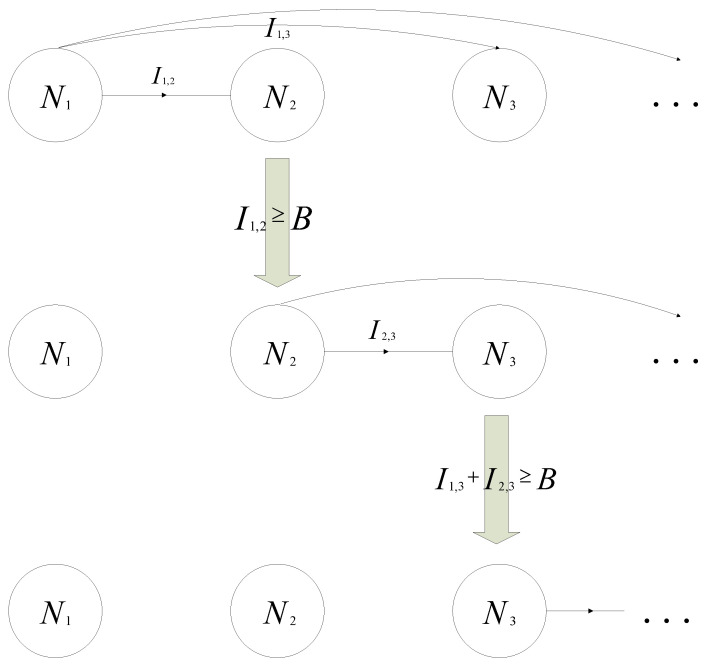
Mutual information accumulation.

The sink node, sensor nodes, and mobile robot all communicate wirelessly. To avoid transmission conflicts, we assume that only one node can transmit at one time. Due to the broadcast characteristics of wireless transmission, all nodes within the transmission range can receive the transmitted information. Normally, information that has not been successfully decoded in one transmission will be dropped. However, this information will remain for the next decoding when the rateless code is used. According to the characteristics of the rateless code, it can be successfully decoded after a sufficient amount of independent information is accumulated from multiple sources. Therefore, for node $N_{j}$, the judgment condition for successful decoding is as follows:

(1) $$\begin{array}{cc} \; & \sum_{i=1}^{j-1}I_{i,j}\geq B, \end{array}$$

where $I_{i,j}$ is the information that $N_{j}$ accumulated from $N_{i}$. *B* is the threshold of the amount of information that ensures successful decoding. After decoding is completed, the node will use independent random number seed encoding and broadcast. Figure 4 shows the transmission process of the first three nodes. The decoding order is <$N_{1}$, $N_{2}$, $N_{3}$>. Initially, $N_{1}$ was broadcasting information. When the information $I_{1,2}$ received by $N_{2}$ and reaches the threshold *B*, $N_{1}$ stops broadcasting and $N_{2}$ starts broadcasting. When the sum $I_{1,3}+I_{2,3}$ received by $N_{3}$ reaches the threshold *B*, $N_{3}$ starts broadcasting and $N_{2}$ stops broadcasting.

According to Shannon’s theorem, $I_{i,j}$ can be obtained as follows:

(2) $$\begin{array}{cc} \; & I_{i,j}=\Delta t_{i}*r_{i,j},\\ \; & r_{i,j}=W*log_{2}\left(1+\lambda_{i,j}\right), \end{array}$$

where $r_{i,j}$ is the information accumulation rate, *W* is the bandwidth, $\lambda_{i,j}$ is the signal-to-noise ratio and $\Delta t_{i}$ is the transmission time of node $N_{i}$.

The transmission delay is determined by the set of nodes participating in the transmission, the decoding order and the transmission time of each participating transmission node. The nodes in the set will be transmitted sequentially according to the decoding order. Each node must have accumulated enough information to decode before starting transmission. The transmission time of each node is greater than or equal to 0. For a given transmission set $V^{\prime }$ and decoding order, the transmission delay is the sum of the transmission time of each node. The minimum delay optimization problem can be expressed as follows:

(3) $$\begin{array}{cc} \min\; & T_{tot}=\sum \Delta t_{i}\\ s.t.\; & \sum_{j=0}^{i-1}\Delta t_{j}*r_{i,j}\geq B\\ \; & \Delta t_{i}\geq 0, \end{array}$$

where $\Delta t_{i}$ is the transmission time of node $N_{i}$. This is a linear problem, but there are $2^{n}$ choices of subsets, and there are n! decoding orders for each subset. It has been proven in [27] that for a specific subset $V^{\prime }$, the optimal decoding order can be obtained by the greedy algorithm. However, we still need to calculate $2^{n}$ kinds of subsets to obtain the optimal solution, which cannot be completed in polynomial time. In addition, this problem has been proven to be NP-complete in [27,31,32]. For mobile robots, the topology and link environment will change quickly. Therefore, the optimal solution that cannot be found in polynomial time is meaningless.

## 4. Dynamic Cooperative Transmission Algorithm

The challenge to algorithm complexity mainly comes from the mobility of mobile robots. When the topology of the network changes due to the movement of robots, the transmission path obtained before will not work. Therefore, we need a dynamic algorithm to obtain a new efficient routing path after the mobile robot moves. It is NP-complete to solve the optimal algorithm for cooperative transmission with mutual information accumulation [27,32], so a heuristic suboptimal algorithm will be proposed in this paper.

In this section, we propose a two-step dynamic cooperative transmission algorithm, which can quickly obtain an efficient routing path. This section is divided into three parts. The first part is the routing path planning of WSNs, the second part is the routing algorithm from the sink node to the mobile robot, and the last part is the routing algorithm from the mobile robot to the sink node.

### 4.1. Fixed Deployment Node Routing Algorithm

In this subsection, we propose a low-delay algorithm from the sink node to all sensor nodes. As mentioned earlier, the optimal algorithm cannot be completed in polynomial time. So we make an assumption for the heuristic algorithm: Let PATH(i) be the optimal routing path from the sink node to node $N_{i}$. Without loss of generality, we assume that node $N_{1}$ is the sink node. For the optimal routing path PATH(j) of node $N_{j}$, the previous hop is node $N_{i}$, then $PATH\left(j\right)=PATH\left(i\right)+\left\{N_{j}\right\}$. Considering the accumulation of information, this assumption may not be correct. However, it is correct without information accumulation. So we use it to design our heuristic algorithm.

We use a modified Dijkstra algorithm to design our heuristic algorithm. The main method of the algorithm is still to determine the path of the nodes one by one until all the node paths are determined, and the algorithm ends. Let $V_{1}$ be the set of decoded nodes, and $V_{2}$ is the set of undecoded nodes. The sink node is initially in $V_{1}$. In each iteration of the algorithm, we will select the node $N_{i}$ with the lowest delay from $V_{2}$, determine its path, and move it to $V_{1}$. After that, we update the time delay of all nodes in $V_{1}$ while the previous hop is $N_{i}$.

However, unlike the conventional Dijkstra algorithm, the delay calculation cannot simply add the delay of each hop separately. Calculating the time delay of each node needs to consider that the past transmission path affects the information accumulation of the current node. The time delay $t_{i}$ when the previous hop of node $N_{i}$ is node $N_{j}$ can be expressed as follows:

(4) $$\begin{array}{cc} \; & t_{i}=t_{j}+\left(B-\sum I\right)/r_{i,j}, \end{array}$$

where $t_{j}$ is the delay of node $N_{j}$ and ∑I is the information accumulated by node $N_{i}$ in the path from the sink node to node $N_{j}$.

Let A be the expected delay matrix, where a(i,j) is the delay of node $N_{i}$ when the previous hop is $N_{j}$. Let T be the transmission time matrix, where t(i,j) denotes the transmission time for the $j_{th}$ hop of the path from the sink node to node $N_{i}$. According to Equations (1) and (2), we can obtain the initial state of matrix A as follows:

(5) $$a\left(i,j\right)=\left\{\begin{array}{cc} 0, & i=1\\ \frac{B}{W*log_{2}\left(1+\lambda_{i,j}\right)}, & j=1\;and\;\lambda_{i,1}>0\\ \infty , & j>1\;or\;\lambda_{i,j}=0 \end{array}\right..$$

In the iterative phase of the algorithm, we find the node in $V_{2}$ with the shortest delay according to matrix A. The nodes $N_{i_{0}},N_{j_{0}}$ with the smallest a(i,j) are found where $N_{i_{0}}$ belongs to $V_{2}$ and $N_{j_{0}}$ belongs to $V_{1}$. $N_{i_{0}}$ is the node with the shortest delay in the current $V_{2}$ and will be moved to $V_{1}$. $N_{j_{0}}$ is the previous hop node of $N_{i_{0}}$. According to the previous assumption, the path from the sink node to $N_{i_{0}}$ is composed of the path from the sink node to $j_{0}$ plus $N_{j_{0}}$ to $N_{i_{0}}$. The path from the sink node to node $N_{i_{0}}$ is recorded and the transmission time of each hop on the path is recorded in matrix T. After that, we need to update all $a(i,i_{0})$ and min(a(i,j)), where $N_{i}$ is in $V_{2}$ according to Equation (4). The transmission time from node $N_{i_{0}}$ to node $N_{i}$ is as follows:

(6) $$\begin{array}{c} ▵t_{i_{0},i}=\frac{B-\sum_{k}t\left(i_{0},k\right)*W*log_{2}\left(1+\lambda_{i,k}\right)}{W*log_{2}\left(1+\lambda_{i_{0},i}\right)}, \end{array}$$

thus, $a(i,i_{0})$ can be updated as follows:

(7) $$\begin{array}{cc} \; & a\left(i,i_{0}\right)=▵t_{i_{0},i}+t_{i_{0}},\\ \; & t_{i_{0}}=\sum_{k}t\left(i_{0},k\right), \end{array}$$

then, continue the iterative process until there are no nodes in $V_{2}$. The complete algorithm is described in Algorithm 1.
**Algorithm 1** Fixed deployment node routing algorithm**Require:** accumulated rate matrix C**Ensure:** static route path PATH  1:Let *V* be the set of all nodes, $V_{1}$ be the set of decoded nodes, and $V_{2}$ be the set of undecoded nodes. Initially, $V_{1}=\left\{N_{1}\right\}$ and $V_{2}=V-\left\{N_{1}\right\}$;  2:Let A be the expected decoding time matrix, where a(i,j) is the decoding time of node $N_{i}$ when the last hop is $N_{j}$. Initialize A through Equation (5);  3:Let T be the transmission time matrix, where $t_{i,j}$ is the *j*th hop transmission time to node $N_{i}$. T is zero matrix initially;  4:$PATH\left(1\right)=\left\{N_{1}\right\}$;  5:**while**$V_{1}!=V$**do**  6:   **for** $N_{i}$ in $V_{2}$, $N_{j}$ in $V_{1}$ **do**  7:     Find $i_{0}$,$j_{0}$ minimizes a(i,j);  8:   **end for**  9:   Move node $N_{i_{0}}$ from $V_{2}$ to $V_{1}$;10:   $PATH\left(i_{0}\right)=PATH\left(j_{0}\right)+\left\{N_{i_{0}}\right\}$;11:   Record the transmission time in matrix T;12:   **for** $N_{i^{\prime }}$ in $V_{2}$ **do**13:     Update $a(i^{\prime },i_{0})$ through Equations (6) and (7);14:     Update $min(a\left(i^{\prime },j\right))$;15:   **end for**16:**end while**

The time complexity of the fixed deployment node routing algorithm is $O(n^{3})$, and the proof is as follows. During the initialization process, all matrices are of size $O(n^{2})$, and the time complexity is $O(n^{2})$. Next is the time complexity of the iterative loop part. Because the $\min(a\left(i^{\prime },j\right))$ for each node $N_{i^{\prime }}$ can be maintained during the initialization process and the subsequent iteration process, only $|V_{2}|$$\min(a\left(i^{\prime },j\right))$ needs to be traversed in the process of finding min(a(i,j)), and the time complexity is O(n). The time to transfer the node from $V_{2}$ to $V_{1}$ is O(1), and the time needed to record the path and time is O(n). In the process of updating $a(i,i_{0})$, according to Equations (6) and (7), each node on the path needs to be calculated once, and the time complexity is O(n). The time for maintenance min(a(i,j)) is O(1). The update needs to be performed on all nodes in $V_{2}$; therefore, the time complexity is $O\left(n\right)*\left(O\left(n\right)+O\left(1\right)\right)=O\left(n^{2}\right)$. The iterative part will be performed n−1 times, so the total time complexity of Algorithm 1 is $O\left(n^{2}\right)+\left(n-1\right)*O\left(n^{2}\right)=O\left(n^{3}\right)$.

### 4.2. Sink to Mobile Robot

In this subsection, we discuss the dynamic routing algorithm from the sink node to the mobile robot. Considering that the request–response process between the mobile robot and the cloud takes a short time and the mobile robot has a limited moving speed, the spatial position of the mobile robot does not change much during this period, so the routing path from the sink node to the mobile robot only needs to follow the reverse path from the mobile robot to the sink node. Since the mobile robot always has to periodically report its location and link status to the cloud due to various requirements, this means that when the cloud wants to send data to the mobile robot directly, it can find the static routing path from the sink node to the mobile robot through Algorithm 1. However, the neighboring nodes of the mobile robot and the link status between the mobile robot and them may still change. The static routing path from the sink node to any sensor node has been obtained above, and then the routing path is dynamically selected based on the neighbor information of the mobile robot. Similar to Algorithm 1, different routing paths will be obtained according to the selection of the previous hop. Therefore, we traverse the neighbors around the mobile robot to find the routing path with the lowest delay.

However, there are two problems with this. The first problem is that the mobile robot may complete information accumulation before the selected neighbor node. In this case, nodes whose decoding time is later than the mobile robot will be removed from the routing path. When the mobile robot finishes data accumulation before selecting neighboring nodes, it will notify the surrounding nodes to stop broadcasting. Another problem is time complexity. We assume that *m* is the number of neighbor nodes of the mobile robot. Because at most *m* nodes on a routing path allow the mobile robot to receive information during transmission, the time complexity of calculating the transmission delay of a routing path is O(m). The time complexity of the path represented by all neighbor nodes is $O(m^{2})$. To reduce the time complexity, we consider only traversing at most *K* neighbor nodes with the best link quality with the mobile robot. Considering that selecting only *K* neighbors for dynamic routing could lead to locally optimal paths, the algorithm can reduce the probability of local optimal occurrence by setting a larger *K* value. The specific value of *K* can be obtained by comparison in experiments. The complete algorithm is described in Algorithm 2.
**Algorithm 2** Dynamic path algorithm**Require:** static route path PATH through Algorithm 1, robot’s one-hop link information, upper limit *K***Ensure:** dynamic route path  1:**while** Initially or robot movement changes the node of the best link **do**  2:   **if** K=1 **then**  3:     Select the node $N_{i}$ with the best link quality with the robot;  4:     Select PATH(i)+{dst} as the dynamic route path;  5:     **if** The robot decodes successfully before node $N_{i}$ **then**  6:        Drop the undecoded node from the dynamic route path;  7:     **end if**  8:   **else**  9:     **for** $N_{i}\in V^{\prime }$, $V^{\prime }$ is the set of *K* neighbors with the best link quality with the mobile robot **do**10:        Select PATH(i)+{dst} as route path;11:        **if** The robot decodes successfully before node $N_{i}$ **then**12:          Drop the undecoded node from the route path;13:        **end if**14:        Calculate the delay of route path *t* through Equations (6) and (7);15:        **if** t<min(t) **then**16:          min(t)=t;17:          Select the route path as the dynamic route path;18:        **end if**19:     **end for**20:   **end if**21:**end while**

Obviously, the time complexity of calculating *K* paths is O(Km). Specifically, when *K* is 1, there is no need to calculate the delay for comparison, and the time complexity is O(1).

### 4.3. Mobile Robot to Sink

In this subsection, we discuss the scenario of sending messages from the mobile robot to the sink node. For cooperative transmission with two fixed nodes, the reverse transmission from the receiver to the sender only needs to exchange the identities of the sender and the receiver and use the algorithm again. However, in the mobile robot scenario, repeated calls to Algorithm 1 will result in high time complexity. In the process from the sink node to the mobile robot, the transmission process is fixed among WSNs. However, the situation is different when the mobile robot is the sender. Due to the different positions of the mobile robot during the transmission from the mobile robot to the sink node, the amount of information accumulated by each node will also vary.

**Figure 5:**
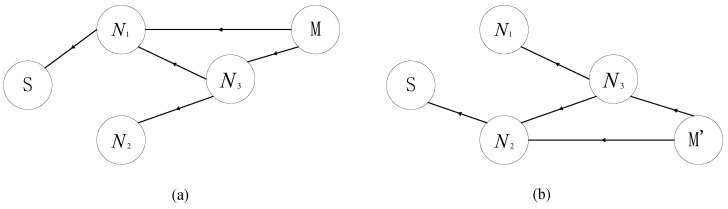
Mobile robot to sink. Decoding order: (**a**) <$M,N_{3},N_{1},S$>; (**b**) <$M^{\prime },N_{3},N_{2},S$>.

As shown in Figure 5, even if the first decoded node is the same, different positions of the mobile robot will still result in different routing paths. In the first case, $N_{1}$ has accumulated a certain amount of information before $N_{3}$ is sent, so it can complete decoding before $N_{2}$. When the mobile robot moves, $N_{2}$ will replace $N_{1}$ to accumulate information in advance and complete the decoding before $N_{1}$. Therefore, Algorithm 2 does not work in this case. If Algorithm 1 is used to solve the optimal routing path, a calculation of $O(n^{3})$ time complexity must be performed every time the mobile robot moves. This is much larger than the O(Km) time complexity of Algorithm 2.

Consider that after the sensor node increases closer to the sink node completes the decoding, the robot’s movement has nothing to do with the subsequent transmission process. We use a greedy distributed algorithm to solve this problem. Similar to opportunistic routing in WSNs, the node with higher priority that completes the decoding first will continue to broadcast as a relay node [33]. How to determine the priority of nodes is a problem. In opportunistic routing, the priority is usually the physical distance to the sink node, the expected delay, or the expected energy consumption, depending on the optimization goal. Considering the delay sensitivity of mobile robots, the priority we choose is the transmission time from the sink node to the node obtained in Algorithm 1.

Initially, the mobile robot broadcasts the information, and all neighboring nodes receive it and try to decode it. The mobile robot stops broadcasting after any neighbor node finishes decoding, and the neighbor node continues broadcasting. If there are multiple neighbor nodes that complete decoding at the same time, the neighbor with the lowest delay in the result of Algorithm 1 will broadcast. The broadcasting node will stop broadcasting until there is a node with lower delay or sink node decoding is completed. The sensor node that receives the information will compare the delay between itself and the sender. If its own delay is lower, it will try to collect enough information to decode; otherwise, it will ignore the transmission. The complete algorithm is shown in Algorithm 3.

Due to the use of the results of Algorithm 1, the distributed heuristic algorithm of Algorithm 3 does not need to calculate the delay again, and the time complexity is O(1).

It is worth noting that the greedy strategy adopted by Algorithm 3 could be prone to local optima and potentially lead to suboptimal paths over time. This problem can be alleviated by setting a reasonable routing algorithm update period. The algorithm provides such a mechanism to set a reasonable update period according to the actual situation, so that the multi-hop relay can be as close to the optimal path as possible and avoid performance degradation as much as possible.
**Algorithm 3** Distributed heuristic algorithm**Require:** transmission time T through Algorithm 1**Ensure:** dynamic route path  1:The mobile robot broadcasts until one of its neighbors finishes decoding and is selected to broadcast information  2:For all sensor nodes:  3:**while** Node $N_{i}$ is selected to broadcast information **do**  4:   Node $N_{i}$ broadcasts information;  5:   **if** node $N_{j}$ finishes decoding and $t_{j}<t_{i}$ **then**  6:     Choose node $N_{j}$ to broadcast information;  7:     break;  8:   **end if**  9:**end while**10:Transmission ends while the sink node decodes

## 5. Performance Evaluation

We simulated our dynamic cooperative transmission algorithm with mutual information accumulation with MATLAB R2020b. The size of the field is set to 90 m × 90 m, and 100 sensor nodes are randomly distributed in the field. The sink node is fixed at coordinates (1,1). The mobile robot is assumed to transport goods between two points, and it moves between two points along a straight line. The moving speed of the mobile robot is set to 1 m/s.

The signal-to-noise ratio in Equation (6) is calculated by the log shading model, which is closer to reality than the Rayleigh model [34]. The signal-to-noise ratio λ(d) can be obtained as follows:

(8) $$\begin{array}{c} \lambda \left(d\right)=P_{t}-\left(PL\left(d_{0}\right)+40log_{10}\left(\frac{d}{d_{0}}\right)+X_{\sigma }\right)-P_{n}, \end{array}$$

where *d* is the distance between two nodes, $P_{t}$ is the transmitting power, $d_{0}$ is a reference distance, $PL(d_{0})$ is the signal strength at $d_{0}$, $X_{\sigma }$ is a random variable with Gaussian distribution with mean 0 and standard deviation σ, and $P_{n}$ is the noise floor. We set $P_{t}=0$ dB, $d_{0}=1$ m, $PL(d_{0})=55$ dB, σ=4 and $P_{n}=-115$ dB. The bandwidth W is set to 10 KHZ, and the threshold B is set to 100 bytes.

### 5.1. Fixed Deployment Node Routing Path Comparison

In this subsection, we will compare our fixed deployment node routing algorithm with the two heuristic algorithms in [27]. In the heuristic Algorithm 1 of [27], only the information transmission of the last hop is considered, and the information accumulated during the transmission of other nodes is ignored. At this time, the problem degenerates into a common single-source shortest path problem, which can be solved directly with the Dijkstra algorithm. Heuristic Algorithm 2 is based on the path of heuristic Algorithm 1, and each time, it searches for whether other nodes have completed decoding and have better link quality to the next hop node. If so, it is added to the path. Our fixed deployment node routing algorithm to find the fixed deployment node routing path has been considered with the entire path information accumulated on the subsequent nodes.

**Figure 6:**
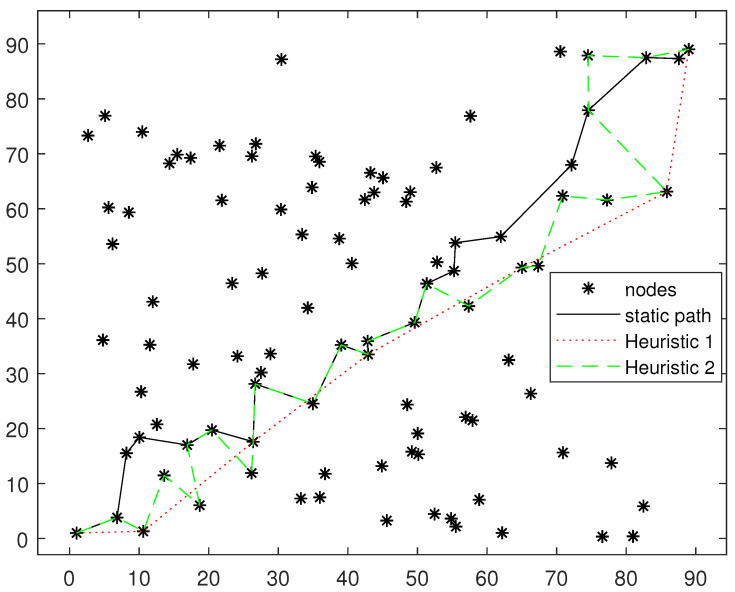
Fixed deployment node routing path comparison. Topology 1.

**Figure 7:**
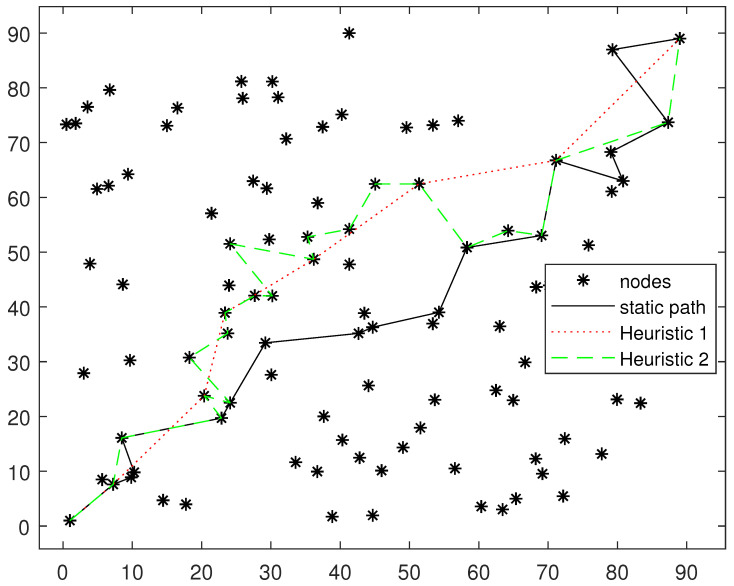
Fixed deployment node routing path comparison. Topology 2.

Figure 6 and Figure 7 show the path selection results of different algorithms on two randomly generated topologies. It can be seen that the distance of each path is relatively long and the number of hops is small in heuristic Algorithm 1. This is because heuristic Algorithm 1 does not consider the accumulation of information for node cooperation. Without considering the accumulation of information, selecting a distant node for direct transmission has a shorter transmission delay because of the lower number of hops. However, if considering the information accumulation, the conclusion will be just the opposite, because long-distance transmission cannot make use of information accumulation. It can also be seen from the figure that the path selected by heuristic Algorithm 2 has passed all the nodes on the path of heuristic Algorithm 1. Based on the path of heuristic Algorithm 1, heuristic Algorithm 2 adds several nodes on both sides of the path into the path. In addition, the fixed deployment node routing algorithm is more inclined to choose continuous and tight routing paths.

**Figure 8:**
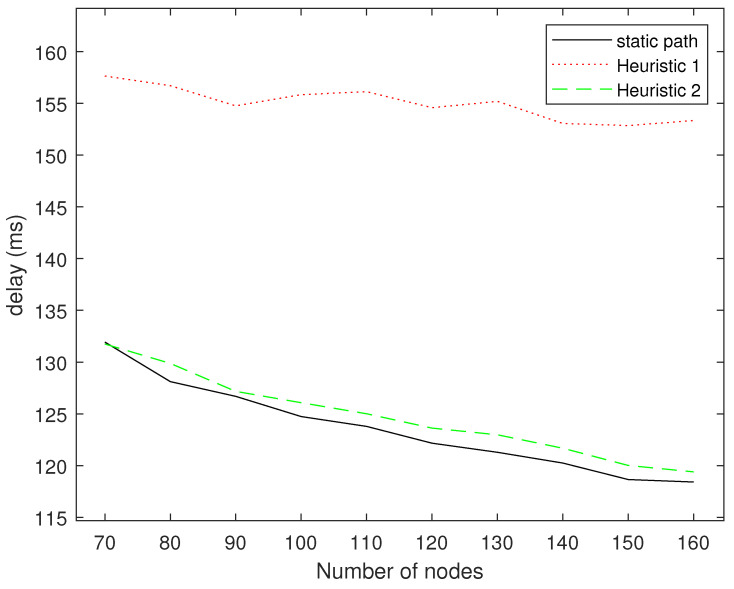
Delay comparison for different numbers of nodes.

Figure 8 shows a comparison of the delays of the three algorithms. We fixed the starting point at (1,1) and the end point at (89,89). For each number of nodes, 50 topological structures were randomly generated, and the results were averaged. The heuristic Algorithm 1 has the highest delay because it does not consider the accumulation of information and the distance between each hop is relatively large. Therefore, the accumulation of information across two hops is often too far away to accumulate information, and the performance of heuristic Algorithm 1 will be close to the transmission without cooperative transmission. Both heuristic Algorithm 2 and our fixed deployment node routing algorithm take into account the accumulation of information between nodes, so the delay is lower. In addition, our algorithm is slightly better than heuristic Algorithm 2.

### 5.2. Dynamic Routing Simulation

**Figure 9:**
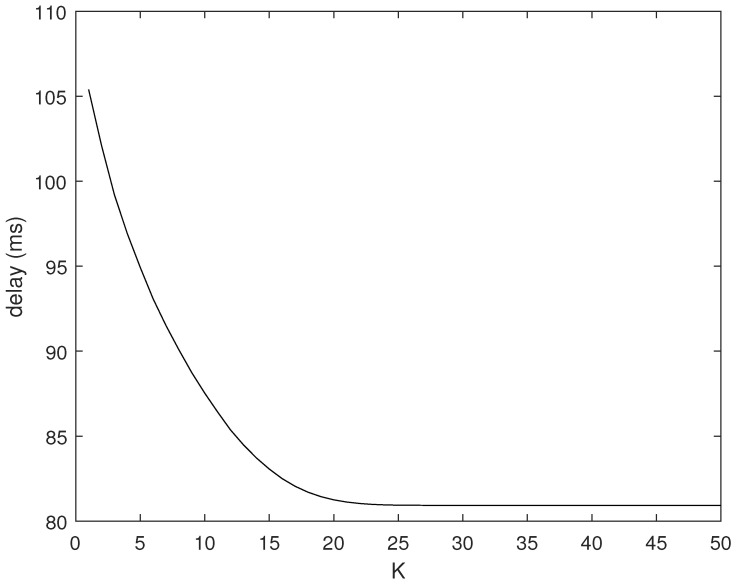
Delay vs. K graph.

In this subsection, we verify the impact of different *K* values on the delay in dynamic path selection through experiments. We repeated the test 100 times for each of the 20 randomly generated topologies. The starting point and ending point of the robot carrying goods are set at (89, 89) and (89, 1), respectively. As mentioned above, to reduce the upper limit of the complexity of our dynamic cooperative routing algorithm, we set an upper limit *K* on the number of neighbors of the mobile robot to be searched. However, without an exhaustive search, it is difficult for us to obtain the optimal solution. Figure 9 shows the impact of different values of *K* on the latency from the sink node to the mobile robot. As shown in Section 4.2, when *K* = 1, it means that only the mobile robot accumulates data from a certain optimal neighbor node. However, this does not take into account that the mobile robot may also accumulate data from other neighboring nodes. When there are multiple nodes around the mobile robot, as the value of *K* increases, Algorithm 2 continuously explores different neighbor nodes as the previous hop node of the mobile robot to determine whether a lower end-to-end latency can be obtained. As shown in Figure 9, when K≤15, the delay of the dynamic cooperative transmission algorithm decreases as the value of *K* increases. When K>15, the reduction in delay gradually slows down and stabilizes.

The reason for this result is the limitation of the number of neighbor nodes around the node. The mobile robot can have 15 neighbor nodes during most of its transport process. The more neighbor nodes it searches, the lower the delay of the best path it can find. When *K* is greater than 15, the mobile robot does not have *K* neighbor nodes during some time periods. Therefore, the probability of finding a better path decreases. When *K* continues to increase, the mobile robot no longer has so many neighbor nodes at any time, so the delay remains unchanged.

**Figure 10:**
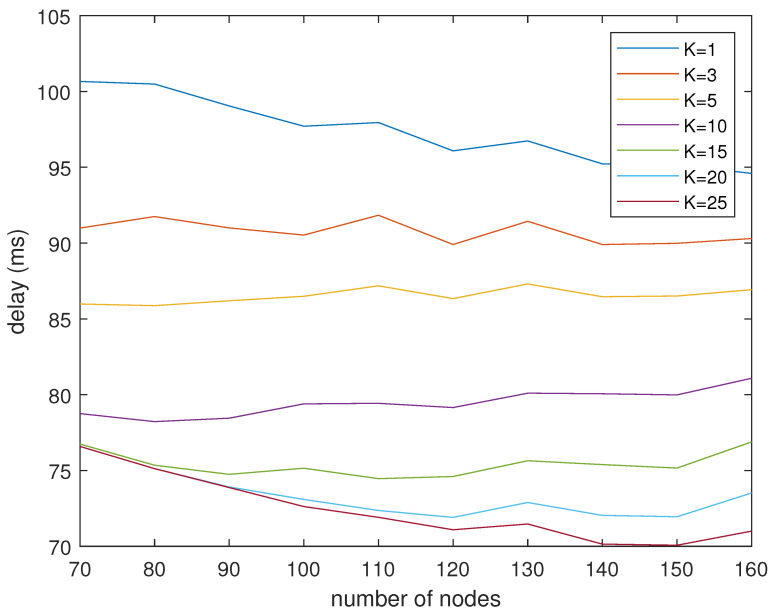
Delay vs. number of nodes graph.

Figure 10 shows the delay of different *K* values under different numbers of nodes. When the number of nodes is small, the delays when *K* is equal to 15, 20, and 25 are almost the same. When the number of nodes increases, the delay of K=20 and K=25 decreases further. This is consistent with the above conclusion that the delay no longer decreases with the increase in *K* due to the limitation of the number of neighbor nodes.

It can be observed that the performance for *K* = 15, 20, 25 is nearly identical with a small number of nodes. When the nodes in the network are dense, assuming that the number of neighbor nodes of the mobile robot is greater than *K*, then the setting of the *K* value will have an impact on the performance of the algorithm. When the nodes in the network are sparse, assuming that the number of neighbor nodes of the mobile robot is less than *K*, the setting of the *K* value will have no impact on the performance of the algorithm.

### 5.3. Mobile Robot to Sink Simulation

**Figure 11:**
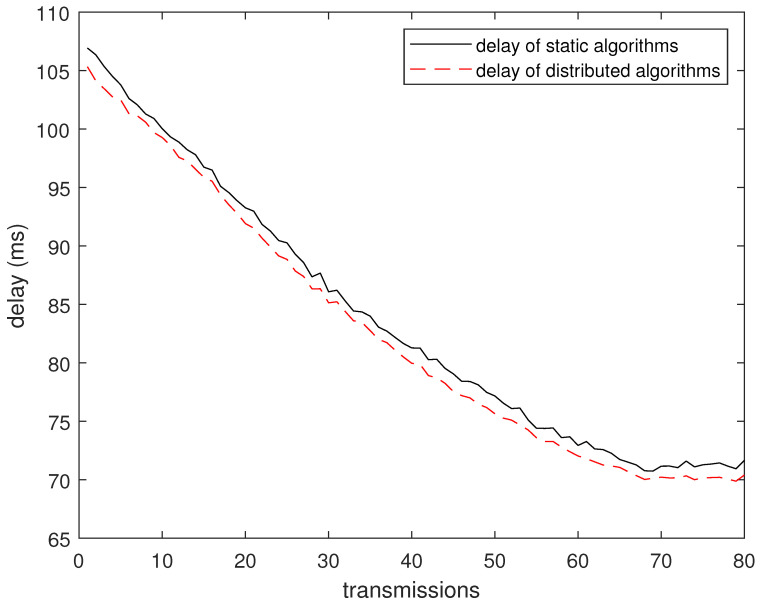
Delay vs. time graph.

In this subsection, the performance of the distributed greedy algorithm is verified through simulation experiments. We compare it with our fixed deployment node routing algorithm, which is proven above to outperform existing heuristic algorithms. The start and end points of the mobile robot are set at (70, 85) and (70, 5), and the robot transmits 80 times to the sink during this period. The experiment randomly generated 10 topological structures and performed 10 times on each topology. As shown in Figure 11, the average delay of the distributed dynamic cooperative transmission algorithm is lower than that of the static route.

**Figure 12:**
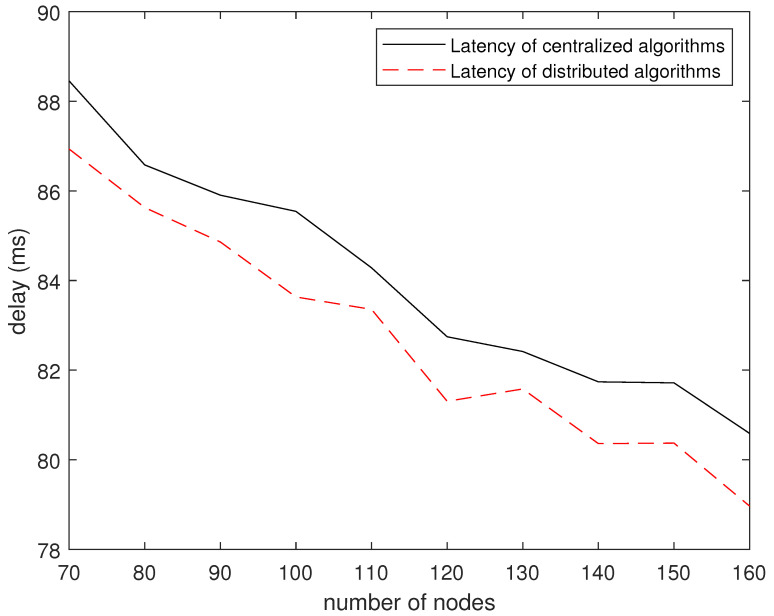
Delay vs. topology graph.

Figure 12 shows the delay of the distributed dynamic collaboration algorithm and the static algorithm under different numbers of nodes. Consistent with previous results, in terms of the number of nodes, the average delay of the distributed dynamic collaboration algorithm is lower than that of the fixed deployment node routing algorithm. As mentioned above, the distributed dynamic cooperative transmission algorithm has lower time complexity when transmitting information from the mobile robot to the fixed node. It can be concluded that the distributed dynamic cooperative transmission algorithm is better in the transmission from the mobile end to the fixed end.

## 6. Conclusions

In this paper, we proposed a dynamic cooperative transmission algorithm to meet the requirements of mobile robots and cloud communication in the IIoT. Since mobile robots are sensitive to time delay, we use cooperative transmission with mutual information accumulation to obtain a lower time delay. Our dynamic cooperative transmission algorithm consists of two parts. The first part is the fixed deployment node routing algorithm to calculate the fixed node delay and path in WSNs. Since these nodes are fixed, the fixed deployment node routing algorithm will only be executed once for a long time, which can accept higher complexity. The second part is dynamic path selection. For the information sent from the cloud to the mobile robot, it selects its own transmission path by searching for the transmission path of neighbor nodes; for the information sent by the robot to the cloud, it dynamically selects the path through a distributed algorithm. The dynamic path selection part will always be carried out when the robot is moving, so the algorithm with lower time complexity is used. The final simulation experiment results show that our algorithm also has good performance in terms of delay.

### 6.1. Limitations

Although simulation experiments have verified the effectiveness of the algorithm proposed in this paper, there are still some potential factors that affect performance in the real world. For example, in complex industrial scenarios, the quality of wireless channels will be affected by the complex and dynamically changing environment and become difficult to predict. Increasing the update frequency of the routing algorithm for fixed deployment nodes can alleviate this problem to a certain extent. However, the dynamic changes in the environment are usually difficult to predict, and the routing algorithm always needs to face the challenge of hysteresis.

### 6.2. Future Research Directions

In this paper, we use Shannon’s theorem (Equation (2)) to obtain a theoretical upper bound on the information accumulation rate, without considering the specific packet size, rateless coding scheme and the probability of packet loss during the transmission of the coded packets. However, rateless coding will introduce additional time overhead, so future research is necessary to explore efficient rateless coding schemes suitable for industrial scenarios in order to strike a balance between decoding delay and the time consumption of encoding data. In addition, it is also helpful to analyze the impact of packet loss (and other possible uncertainties) on the performance of the algorithm.

Due to the length and subject limitation, this paper abstracts the research problem into a multi-hop routing problem between a single mobile robot and a single sink node pair, without involving multiple mobile robots. In order to further improve the practicality and performance of the proposed algorithm, it is also necessary to explore suitable MAC protocols to cope with collision-free concurrent transmission between multiple mobile robots and sink nodes.

## Data Availability

The original contributions presented in the study are included in the article. Further inquiries can be directed to the corresponding authors.
